# Heparanase inhibition preserves the endothelial glycocalyx in lung grafts and improves lung preservation and transplant outcomes

**DOI:** 10.1038/s41598-021-91777-0

**Published:** 2021-06-10

**Authors:** Kentaro Noda, Brian J. Philips, Mark E. Snyder, Julie A. Phillippi, Mara Sullivan, Donna B. Stolz, Xi Ren, James D. Luketich, Pablo G. Sanchez

**Affiliations:** 1 Division of Lung Transplant and Lung Failure, Department of Cardiothoracic Surgery, University of Pittsburgh, UPMC Presbyterian C-900, 200 Lothrop St., Pittsburgh, PA 15213 USA; 2 Division of Pulmonary, Allergy and Critical Care Medicine, Department of Medicine, University of Pittsburgh, Pittsburgh, USA; 3 Division of Cardiac Surgery, Department of Cardiothoracic Surgery, University of Pittsburgh, Pittsburgh, USA; 4 Department of Bioengineering and McGowan Institute for Regenerative Medicine, University of Pittsburgh, Pittsburgh, USA; 5 Department of Cell Biology and Physiology, University of Pittsburgh, Pittsburgh, USA; 6 Department of Biomedical Engineering, Carnegie Mellon University, Pittsburgh, PA USA

**Keywords:** Glycobiology, Experimental models of disease, Preclinical research, Acute inflammation, Organ transplantation, Translational research, Extracellular matrix, Cell invasion

## Abstract

The endothelial glycocalyx (eGC) is considered a key regulator of several mechanisms that prevent vascular injury and disease. Degradation of this macromolecular layer may be associated with post-transplant graft dysfunction. In this study, we aimed to demonstrate the benefits of eGC protection via heparanase inhibition on graft quality. We established rat models of lung grafts with damaged or preserved eGC using ischemic insult and transplanted the grafts into recipients. Lung grafts were also subjected to normothermic ex vivo lung perfusion for detailed assessment under isolated conditions. Physiologic parameters and eGC-associated cellular events were assessed in grafts before and after reperfusion. Structurally degraded eGC and highly activated heparanase were confirmed in lungs with ischemic insult. After transplant, lungs with damaged eGC exhibited impaired graft function, inflammation, edema, and inflammatory cell migration. Increased eGC shedding was evident in the lungs after reperfusion both in vivo and ex vivo. These reperfusion-related deficiencies were significantly attenuated in lungs with preserved eGC following heparanase inhibition. Our studies demonstrated that eGC plays a key role in maintaining lung graft quality and function. Heparanase inhibition may serve as a potential therapeutic to preserve eGC integrity, leading to improved post-transplant outcomes.

## Introduction

The endothelial glycocalyx (eGC) is a complex matrix of membrane-bound proteoglycans, glycoproteins, glycosaminoglycans (GAGs), and adherent plasma proteins that lines the internal surfaces of blood vessels^1–3^. This protective layer shields the entire endothelial luminal surface, reduces the shear stress of blood flow, and regulates platelet adhesion, inflammatory cell migration, and water penetration^4^. Under normal physiological conditions, the integrity of the eGC is well-maintained in a healthy endothelial vascular lumen. In contrast, under stressed conditions, eGC degradation can rapidly occur via enzymatic induction by active heparanase, an endogenous β-d-glucuronidase^5,6^. Because eGC breakdown and shedding results in endothelial barrier dysfunction, reduced nitric oxide synthesis, and cellular invasion, it is believed to be a major trigger of microvascular damage. Indeed, organ failure and the development of vascular diseases are known to be directly linked with eGC degradation^4,7^.

During lung transplantation, signaling pathways inducing microvascular damage likely contribute to poor graft quality, primary graft dysfunction, and acute cellular rejection and could compromise posttransplant outcomes^8,9^. The importance of endothelial preservation in grafts is widely accepted in the transplant community^10^. Because the eGC is highly susceptible to cellular stress, identifying cellular and molecular targets that preserve eGC integrity may also lead to improved endothelial function and posttransplant outcomes.

In this study, we hypothesized that increased eGC degradation may critically impact graft quality and attenuate post-transplant graft functional outcomes. To address this hypothesis, we established rat lung models with damaged or preserved eGC and tested lung graft function after single-lung transplant or ex vivo lung perfusion (EVLP).

## Results

### Confirmation of an established intragraft endothelial glycocalyx damage model

**Figure 1:**
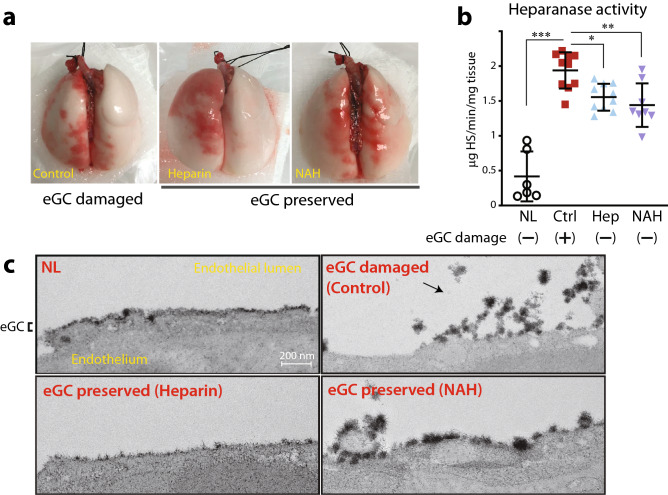
(**a**) Representative images of rat lungs after procurement. (**b**) Heparanase activity was detected in tissue after procurement following 1 h of warm ischemia. (**c**) Ultrastructural images of the endothelial glycocalyx (eGC) from lung grafts after 1 h of warm ischemia obtained using transmission electron microscopy. The labeled glycocalyx appears as the darker cell surface layer; black arrow indicates glycocalyx desquamated from the surface of an endothelial cell. *p < 0.05, **p < 0.01. *NL* native lungs, *Ctrl* control lungs with eGC damage induced by 1 h of ischemia, *Hep* lungs with eGC preserved by heparin administration prior to 1 h of ischemia, *NAH* lungs with intact eGC preserved by N-acetyl heparin administration prior to 1 h of ischemia.

To perform this study, we first established an animal model of eGC damage in lung grafts induced by ischemic insult in vivo. After cardiopulmonary arrest was induced in donor rats with an anesthesia overdose, the donor was kept at room temperature for an hour to induce ischemic insult in the lungs. In some rats, heparin, a clinically available heparanase inhibitor, was administered intravenously via the jugular vein 5 min before the ischemic insult with the purpose of preserving the eGC from enzymatic degradation by heparanase. Other rats received N-acetyl heparin (NAH), a heparin-derivative that lacks anticoagulant activity, which allowed us to confirm that heparin’s effects on the eGC were independent of its anti-coagulation activity. After 1 h of in vivo ischemic insult at room temperature, lungs were procured in a standardized fashion for lung transplantation. Gross visual inspection of excised lungs revealed no significant changes in appearance following heparin or NAH treatment (Fig. 1a). However, heparanase activity was significantly elevated in lungs with no treatment and was inhibited by both heparin and NAH (Fig. 1b) No differences in heparanase protein expression in the lung tissues were noted among the groups (Supplemental Fig. S1).

Transmission electron microscopy (TEM) revealed ultra-structural changes in the eGC in the lung vasculature after warm ischemic insult. Fixation buffer containing 2% lanthanum, which specifically binds to the glycocalyx, was perfused via the pulmonary artery during graft procurement to label and visualize the eGC under TEM. In control lungs, the eGC was desquamated from the endothelial cell surface with complete dissociation in certain regions. In contrast, the eGC was intact and adherent to the cell surface in lungs pre-treated with either heparin or NAH prior to ischemic insult, similar to the eGC morphology observed in native lungs (Fig. 1c, Supplemental Fig. S2). After establishing these experimental models of lungs with damaged (control; no treatment) or preserved eGC (heparin/NAH treated) regulated by heparanase activity, we began to investigate how the status of intragraft eGC affects post-transplant graft quality in lung transplantation.

### Physiological performance and immune cell infiltration after transplant in lung grafts with damaged endothelial glycocalyx

**Figure 2:**
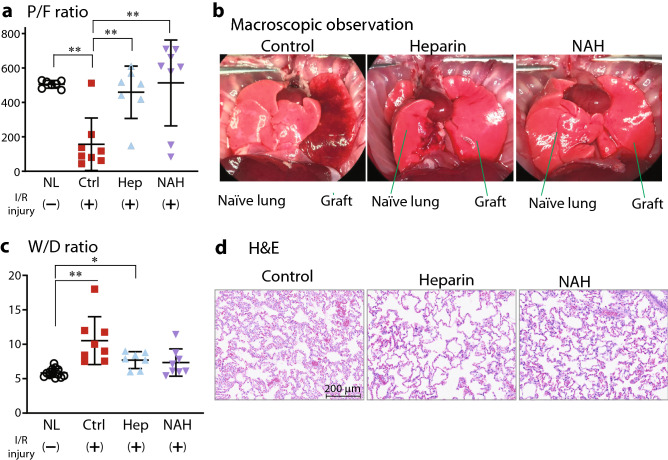
The effect of eGC damage in the lung grafts on the posttransplant graft function and quality. Grafts were evaluated 2 h after transplantation for (**a**) PaO2/FiO2 (P/F ratio), (**b**) macroscopic morphology (representative samples are shown), (**c**) Wet-to-dry (W/D) ratio, and (**d**) microscopic morphology (representative H&E stained samples are shown). *p < 0.05, **p < 0.01. *NL* native lungs, *Ctrl* control lungs with eGC damage prior to transplant, *Hep* lungs with eGC preserved by heparin prior to transplant, *NAH* lungs with eGC preserved by N-acetyl heparin prior to transplant, *I/R injury* ischemia reperfusion injury.

To examine the impact of eGC damage on graft quality after transplantation using our established models, we performed rat orthotopic single lung transplantation using a 3-cuff technique^11,12^. Lung grafts in all groups were subjected to cold ischemia at 4 °C for 6 h, then transplanted into syngeneic recipients and reperfused for 2 h to simulate a clinical scenario. Two hours after reperfusion, the gas exchange ability of the lung grafts was evaluated by gas analysis of blood in the pulmonary vein of the graft during single-lung ventilation with 100% O_2_. Lungs in control exhibited impaired gas exchange function as indicated by a reduced PaO_2_/FiO_2_ (P/F) ratio 2 h after reperfusion, when compared with native lungs. Conversely, lung grafts with preserved eGC due to heparin or NAH treatment displayed markedly improved post-transplant function, as indicated by a significantly higher P/F ratio 2 h after transplantation as compared with control lung grafts (Fig. 2a). Reperfusion-induced pulmonary edema was clearly evident in lungs without heparin/NAH pretreatment, both macroscopically (Fig. 2b) and microscopically (Fig. 2d) and was objectively quantified by measuring the wet-to dry (W/D) ratio. Control lungs after transplant had significant congestion, swollen alveolar walls, and secretions in the airways, and the W/D ratio was twice that observed in native lung samples (p < 0.01) (Fig. 2c). In contrast, grafts with preserved eGC after either heparin or NAH administration exhibited less pulmonary edema, which was reflected in a lower W/D ratio 2 h after transplantation. Significant congestion, swollen alveolar walls, and secretions in the airways were not observed in lungs with preserved eGC after heparanase inhibition by heparin/NAH (Fig. 2d).

**Figure 3:**
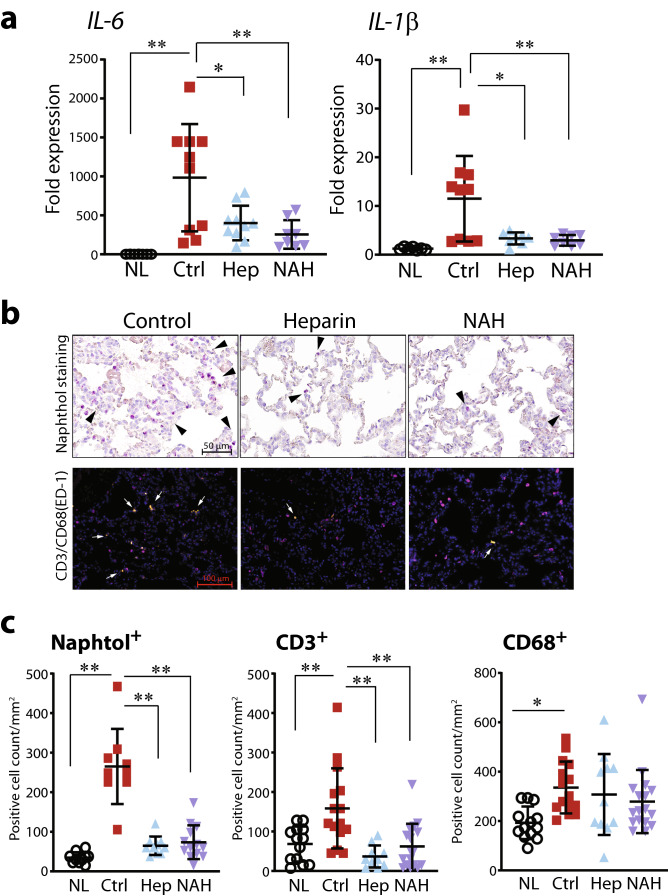
Endothelial glycocalyx influences the inflammation and inflammatory cell migration in lung grafts after transplantation. (**a**) Real-time RT-PCR for the mRNA of proinflammatory cytokines *interleukin (IL)-6 and IL-1β*. (**b**) The extravasation of neutrophils, T cells, and monocytes in the transplanted grafts were evaluated by specific staining of polymorphonuclear neutrophils (naphthol staining, positive cells indicated by black arrow heads), T cells (CD3^+^-positive, indicated by white arrows) and macrophages (CD68^+^-positive, purple staining), and (**c**) quantitated. *p < 0.05, **p < 0.01. *NL* native lungs, *Ctrl* control lungs with eGC damage prior to transplant, *Hep* lungs with eGC preserved by heparin prior to transplant, *NAH* lungs with eGC preserved by N-acetyl heparin prior to transplant.

The expression profiles of mRNAs for proinflammatory cytokines within the graft tissues 2 h after reperfusion were examined using quantitative real-time reverse-transcription polymerase chain reaction (RT-PCR). Proinflammatory cytokine expression of both interleukin *(IL)-6* and *IL-1β* were higher in control lung grafts than in native lungs (Fig. 3a). In contrast, mRNA expression levels of *IL-6* and *IL-1β* were significantly lower in grafts treated with heparin/NAH as compared with lung grafts in control. In addition, we found that reduced cellular infiltration was directly associated with eGC preservation after heparanase inhibition. Specifically, graft-tissue staining indicated significantly reduced polymorphonuclear neutrophils (PMNs) (naphthol-positive staining), T-cell (CD3^+^-positive immunostaining) and macrophage (CD68^+^-positive immunostaining) infiltration in lungs with preserved eGC by heparin/NAH administration as compared with control lung grafts 2 h after reperfusion (Fig. 3b,c).

### Further investigation of eGC shedding and endothelial damage after transplantation in the lungs with intact versus damaged endothelial glycocalyx

**Figure 4:**
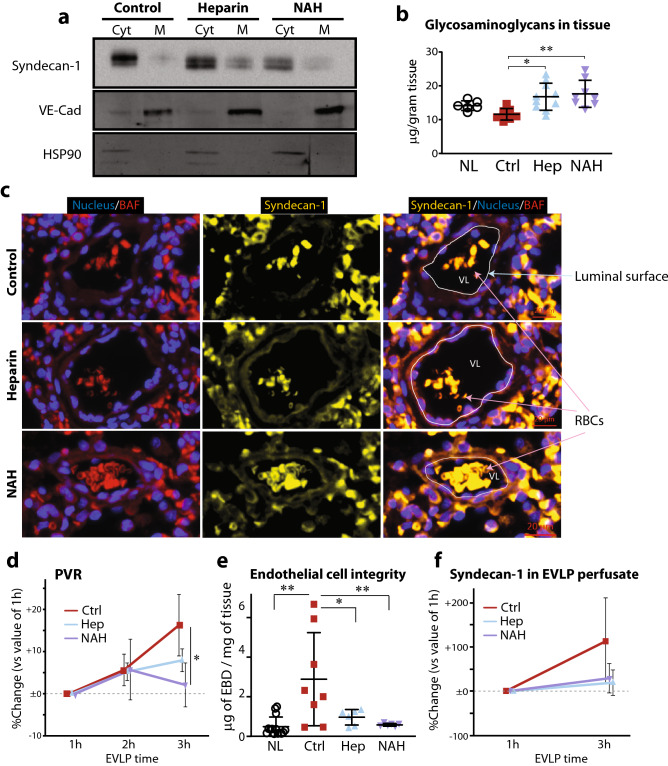
Assessment of endothelial glycocalyx and microvascular integrity in lung grafts after reperfusion. (**a**) Western blot of syendecan-1 in fractionated lung tissue 2 h after transplantation. Gel image depicts a single representative sample from each treatment group. Vascular endothelial cadherin (VE-Cad) and heat shock protein 90 (HSP90) were blotted as loading markers for the plasma membrane and cytosolic protein fractions, respectively. A full-length image of this blot and the blots showing multiple samples for each treatment group are shown in Supplemental Figs. S3–S5. *Cyt* cytosol protein fraction, *M* membrane protein fraction. (**b**) Glycosaminoglycan (GAG) content in the grafts 2 h after transplantation. (**c**) Syndecan-1 staining (yellow) in the peripheral vasculature (φ ~ 50 mm) of the grafts 2 h after transplantation. The endothelial luminal surface is indicated as a white line in the merged images (right column). Nuclei were stained using Hoechst 33342. *BAF* background autofluorescence, *RBCs* red blood cells, *VL* vascular lumen. (**d**) Pulmonary vascular resistance (PVR) of lung grafts during ex vivo lung perfusion (EVLP). Time-dependent fold changes from the values at 1 h are shown. n = 4–5 for each group. (**e**) Endothelial barrier integrity after EVLP was assessed by measuring the amount of Evans blue dye (EBD) retained in the tissue. (**f**) Time-dependent changes of syndecan-1 in the perfusate during EVLP. *p < 0.05, **p < 0.01. *NL* native lungs, *Ctrl* control lungs with eGC damage prior to transplant/EVLP, *Hep* lungs with eGC preserved by heparin treatment prior to transplant or EVLP, *NAH* lungs with eGC preserved by N-acetyl heparin treatment prior to transplant or EVLP.

After demonstrating changes in tissue physiology and extravasation of inflammatory cells in grafts during reperfusion dependent on the integrity of the eGC, we next analyzed eGC integrity in lung grafts after transplantation. Membrane and cytosolic proteins fractions were isolated from lung tissues after transplantation to quantitate the remaining glycoproteins on the plasma membrane by western blotting and compare glycoprotein differences between grafts with and without pretransplant heparanase inhibition. Successful separation and isolation of membrane and cytosolic fractions from tissue samples was confirmed by western blotting for vascular endothelial (VE)-cadherin and heat shock protein 90 (HSP90) as loading markers for plasma membrane protein fractions and cytosol protein fractions, respectively (Supplemental Fig. S3). Syndecan-1 (a major eGC proteoglycan) protein expression in the plasma membrane was markedly higher 2 h after transplantation in heparin/NAH treated lung grafts as compared with control lung grafts (Figs. 4a, Supplemental Figs. S4, S5). Similarly, 1,9-dimethylmethylene blue (DMMB)-based GAG quantification assays revealed that significantly more GAGs remained in the lung tissue 2 h after transplant in grafts with heparin/NAH preserved eGC as compared with control lungs grafts with no eGC protection (Fig. 4b). Immunofluorescent (IF) staining for syndecan-1 in lung grafts 2 h after reperfusion was performed to assess localization of syndecan-1 in peripheral vasculature and airway and to clarify whether syndecan-1 was lost from the vascular endothelium. Staining was assessed in the peripheral lung vasculature (φ ~ 50 μm) and in the pulmonary epithelium in the respiratory bronchioles near the alveolar ducts (φ ~ 100 μm). IF staining clearly showed loss of syndecan-1 expression in the endothelial lumen of the peripheral vasculature in the transplanted control lung tissue with no protection of eGC as compared with transplanted lungs with preserved eGC (heparin or NAH-treated) (Fig. 4c). In contrast, syndecan-1 was intact on the endoluminal portion of the pulmonary epithelium where respiratory bronchioles transitioned to alveolar ducts in the transplanted lung tissue, and no difference was observed among treatment groups (Supplemental Fig. S6).

To investigate the dynamic changes in the vasculature and endothelial barrier function of lungs during reperfusion in an isolated environment, we performed normothermic acellular EVLP on the rat lungs. To specifically focus on the effects of eGC status, we minimized cold ischemic time to 1 h before placing the lung grafts to EVLP. Lungs from the damaged-eGC and preserved-eGC rat models were perfused with acellular STEEN solution and ventilated on the system at normothermia for 3 h. The control lungs exhibited a significant increase in pulmonary vascular resistance (PVR) with time during EVLP, while lungs with preserved eGC with donor heparin/NAH treatment had little to no increase in PVR while on EVLP (Fig. 4d). Endothelial barrier integrity was quantified by examining Evans blue dye (EBD) penetration into the lung parenchyma during the last 30 min of EVLP. The lungs in control showed significantly increased penetration of EBD as compared with native lungs. In contrast, both heparin-treated and NAH-treated lungs displayed significantly less EBD penetration (Fig. 4e). Furthermore, we found that syndecan-1 concentrations in the EVLP perfusate were maintained at a constant level during EVLP of lungs with heparin/NAH preserved eGC as compared with increasing levels of syndecan-1 in the perfusate from lungs in control (Fig. 4f).

**Figure 5:**
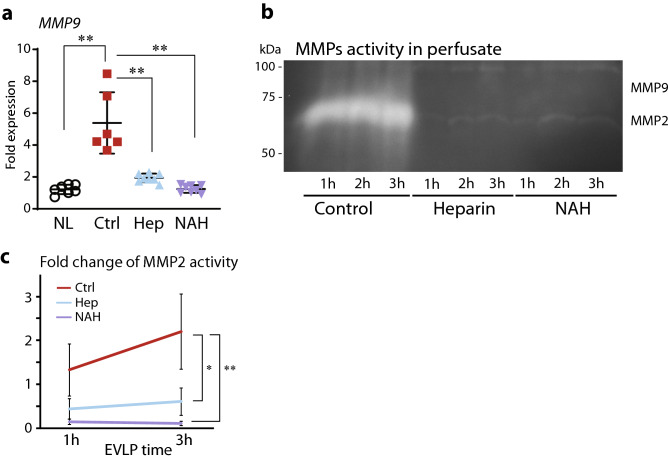
The effects of heparanase inhibition on MMP-2 and MMP-9 activity. (**a**) The mRNA expression of metalloprotease *(MMP)-9* in lung grafts 2 h after transplantation. (**b**) Representative gelatin zymography of time-dependent changes in activity of secreted MMP-2 and MMP-9 in EVLP perfusate. Full length of gels and replications are shown in Supplemental Fig. S7. (**c**) MMP-2 activity quantitated and shown as fold change. *p < 0.05, **p < 0.01. *NL* native lungs, *Ctrl* control, *Hep* heparin, *NAH* N-acetyl heparin.

In addition to heparanase, other proteolytic enzymes play an important role in regulating homeostatic eGC shedding, and MMP-2 and MMP-9 are known to cleave syndecan-1 at the cell membrane. Accordingly, we examined the mRNA expression profiles of MMP-9 in lung tissue after transplant using quantitative RT-PCR. *MMP-9* mRNA expression was significantly lower in grafts treated with heparin/NAH as compared with lung grafts in control 2 h after transplantation (Fig. 5a). To further confirm these findings, we measured secreted MMP-2 and MMP-9 enzymatic activity in the EVLP lung perfusate using gelatin zymography. In control lungs, MMP-2 activity in the perfusate increased significantly over time. In stark contrast, MMP-2 activity in EVLP perfusate from lungs with heparin/NAH pretreated was significantly less than that seen in controls with eGC damage and remained unchanged during EVLP (Fig. 5b,c). The activity of MMP-9, which predominantly exists in inflammatory cells (e.g. neutrophils)^13,14^ was low and did not changed during EVLP, and no significant differences in MMP-9 activity were observed among groups (Fig. 5b). This suggests that MMP-2 may uniquely facilitate eGC shedding after reperfusion. Gelatin zymography using tissue lysates supported this finding and showed that endogenous MMP2 was predominantly expressed in pretransplant lung tissue regardless of the status of the eGC (Supplemental Fig. S7), suggesting that the observed glycocalyx shedding during EVLP primarily resulted from tissue-specific factors. Overall, these findings suggest that donor lungs with preserved eGC display significantly reduced tissue-specific MMP-2 protein activity.

## Discussion

Using in vivo and ex vivo experimental models, this study revealed links between eGC integrity and lung transplant outcomes. We demonstrated that eGC fragility increases the risk of graft failure after reperfusion, which further increases eGC shedding and microvascular damage and dysfunction. Additionally, we showed that grafts with preserved eGC via heparanase inhibition exhibited better post-transplant function and quality. The findings suggest that ischemic insult can trigger significant eGC degradation via heparanase activation, culminating in ultrastructural vulnerability of the eGC and associated decoupling from the endothelium. We observed ultrastructural disruption of the eGC in the endothelial lumen of lungs after ischemic damage using TEM. Furthermore, investigation of the lungs using EVLP demonstrated that significant endothelial dysfunction was associated with eGC damage and shedding even in a simplified ex vivo system. Our findings suggest that heparanase inhibition could provide a novel therapeutic means for pulmonary endothelial protection leading to better graft preservation and post-transplant outcomes.

Breakdown of the eGC has been investigated in the etiology of cardiovascular disease, and increasing evidence has demonstrated a link between eGC shedding and the development of acute and chronic organ failure^2,15^. Because degradation of the eGC is induced by both cellular insults and clinical events, several clinically relevant in vitro and in vivo research models have been established to further study eGC damage using ischemia–reperfusion injury, tumor necrosis factor (TNF)-α administration, oxidative stress, and active eGC degradation enzymes (e.g. heparanase). In this study, we established a lung graft model with damaged eGC using ischemic insult to gain insight into the relationship between eGC stability and post-transplant outcomes. Overall, our findings are consistent with the importance of endothelial protection in all solid organ transplants including the lung.

The mechanisms underlying eGC breakdown and associated physiological outcomes have been increasingly investigated over the last decade. The enzymatic activity of heparanase, which has been identified as a key endoglycosidase responsible for eGC degradation^6^, can be controlled by various therapeutic interventions including heparin and heparinoids, quinolines and aspirin^16^. In recent years, several heparanase inhibitors have been synthesized and are currently being studied in clinical trials as potential cancer treatments^17^. In this study, we utilized heparin to preserve the eGC under ischemic conditions because of its well defined biocompatibility and clinical safety. We demonstrated that heparin and NAH effectively inhibit heparanase activity and protect lung graft function via stabilization of the eGC during the ischemic insult to the donor. Heparin’s inhibitory effect on heparanase was independent from its anticoagulation properties as previously reported^18,19^. The administration of heparin in current clinical practice, typically a single intravenous dose before procurement, is not intended for endothelial or eGC protection, however. Indeed, degradation and shedding of the eGC in organ grafts has been reported in the clinical setting^14,20–23^. Moreover, heparin’s adverse effects and limitations have been confirmed with respect to eGC stability and endothelial function^24–26^. For example, VanTeeffelen and colleagues demonstrated that heparin binding and interaction with glycocalyx-related proteins may impair nitric-oxide-dependent arterial vasodilation and induce vascular leakage of colloid substances^24^. Therefore, using heparin to preserve the glycocalyx may be suboptimal, and other potent heparanase inhibitors and improved endothelial protective strategies are desirable for better organ preservation and better post-transplant outcomes.

Because heparanase is a heparan-sulfate-specific shedding enzyme^27^, our findings suggest an important role of heparan sulfate itself in maintaining eGC stability. In addition to heparanase activity, the overall eGC degradation process undoubtably involves the coordinated activation of multiple specific eGC shedding enzymes^28,29^. In this study, the use of EVLP revealed that activated MMP-2, a key enzyme involved in the proteolytic cleavage of eGC, increased in a time-dependent manner, resulting in prominent GAG and syndecan-1 degradation following reperfusion. Our findings that pre-treatment with heparanase inhibitors effectively blocked GAG and syndecan-1 shedding and MMP-2 activation in lungs during subsequent reperfusion imply that activated heparanase or fragmented heparan sulfate could be a primary driving force for further eGC disintegration^2,30,31^. As such, heparan sulfate may sustain eGC structural integrity, and its degradation could initiate enzymatically-induced eGC desquamation. While our group and others previously demonstrated that passenger leukocytes are circulating in the perfusate during EVLP, MMP-9 activity was not detected in EVLP perfusate in this study. This could be because of the acellular perfusate or dilution effects, and the similar findings were reported by Sladden and colleagues previously^14^. Because MMP-9 is predominantly expressed and released from leukocytes during their extravasation process^13,32^, MMP-9 activity may not be observed in acellular perfusate or cellular perfusate with leukocyte-depleted packed red blood cells, as Sladden et al. found. Our findings suggest that leukocyte extravasation does not occur primarily during the ischemic period in the donor but rather during reperfusion after transplantation. Of note, our study found MMP-9 mRNA upregulation and increased counts of T-cells/neutrophils/monocytes in transplanted grafts with eGC damage, suggesting that leukocyte extravasation may impact eGC degradation during in vivo reperfusion. Clearly, further studies are necessary to elucidate the mechanisms underlying heparanase inhibition and eGC integrity and their impact on organ dysfunction and post-transplant outcomes.

### Limitations

Our studies using a rat lung graft model provide strong evidence for improved post-transplant graft function; however, the rat model has some limitations as described previously^33^. In this study, we focused on acute phase changes after transplantation because these are one of the strengths of the model. Studies involving chronic changes in graft function will likely require a different animal model. Additionally, we measured GAGs using a DMMB quantification assay. This methodology has technical limitations in that it cannot distinguish between endogenous GAGs and exogeneous heparin and NAH, which could potentially remain in tissue even after transplantation. Although it is likely that the heparin and NAH administered were significantly diluted in the donors and then potentially eliminated from the lung tissue through transplant process, we cannot rule out the possibility that the DMMB results may include exogenous heparin or NAH. Further research is needed to develop methods to differentiate between endogenous and exogeneous glycocalyx components.

In summary, eGC vulnerability induced by ischemic damage directly resulted in inflammation, edema, and immune/inflammatory cell migration to the lungs after reperfusion in this rat model. Preserving the eGC by inhibiting heparanase improved graft function and reduced inflammation, suggesting the critical role of eGC in attenuating reperfusion injury in transplanted grafts. Consequently, structural preservation of the eGC may be a key therapeutic strategy to improve post-transplant outcomes.

## Materials and methods

All experimental procedures and protocols were approved by the Institutional Animal Care and Use Committee at the University of Pittsburgh and performed according to their guidelines and the National Research Council’s Guide for the Humane Care and Use of Laboratory Animals. And this study was carried out in compliance with the ARRIVE guidelines (http://www.nc3rs.org.uk/page.asp?id=1357).

### Study design

To investigate the stability of eGC in lung grafts and its potential influence on graft function and quality after reperfusion, we established rat models with damaged or preserved lung eGC and performed orthotopic, single, left-lung transplantation or EVLP using the lungs from these rats. Animals were randomly assigned 4 different groups; (1) native lungs (normal control), (2) Control: lungs with eGC damage, (3) Heparin: lungs with eGC preserved by heparin, and (4) NAH: lungs with eGC preserved by NAH. Shedding of the eGC was induced in vivo by donor systemic ischemia. After 1 h of ischemia in vivo*,* lungs were procured in a standardized fashion for lung transplantation^11,12^ then either directly transplanted or subjected to EVLP and assessed biologically. Native lungs were not subjected to any insult.

### Animals

Inbred male Lewis (RT-1^l^) rats weighing 250–300 g were purchased (Harlan Sprague–Dawley Inc., Indianapolis, IN). Animals were maintained in laminar flow cages in a specific-pathogen-free animal facility at the University of Pittsburgh and fed a standard diet and water ad libitum.

### Models of lungs with damaged or preserved eGC and rat orthotopic left lung transplantation

Rats were sedated with 4% isoflurane via inhalation for tracheostomy and then placed on a ventilator. They received 5% isoflurane with 100% O_2_ via inhalation through the ventilator for 15 min to induce a deep state of anesthesia that causes arrest of spontaneous breathing. The rats were disconnected from the ventilator 5 min after intravenous administration of heparin (300 IU), a heparin derivative without anti-coagulant properties (300 μg; N-acetyl heparin (NAH) (Millipore Sigma, Burlington, MA)), or saline (300 μl, control) through the jugular vein. Cardiac activity and blood oxygen saturation were monitored after disconnection from ventilator until cardiac arrest. One hour after induction of ischemia, a median thoracotomy was performed, and blood was flushed from the lungs with cold, low potassium dextran solution (PERFADEX; XVIVO Perfusion AB) through the pulmonary artery. Then, the heart–lung bloc was isolated and stored in cold PERFADEX for 6 h before transplantation.

Orthotopic single-lung transplantation of the left lung was performed using the cuff method as described previously^11,12^. Two hours after reperfusion, the naïve lung was clamped, 100% O_2_ was administered for 5 min through a ventilator, and the recipient’s blood was sampled from the graft pulmonary vein for blood gas analysis.

### Ex vivo lung perfusion in rats

EVLP was performed using a commercially available rodent EVLP system (IPL-2 Isolated Perfused Rat or Guinea Pig Lung System; Harvard Apparatus, Holliston, MA) as described previously^11^. After procurement, the lungs were kept under cold ischemia for 1 h, then placed on the EVLP system, ventilated with air warmed to 37°C, and perfused with 100 ml STEEN solution (XVIVO Perfusion AB) that was deoxygenated with 6% O_2_, 8% CO_2_ and balanced N_2_ and supplemented with 50 mg of methylprednisolone (Solu-Medrol; Pfizer, Inc.) and 50 mg of cephalosporin (Cefazolin; West-Ward Pharmaceuticals Corp., Eatontown, NJ). Perfusion flow was started at 10% of target flow and gradually increased for 1 h toward a target flow rate that was calculated as 20% of cardiac output (75 ml/min/250 g donor body weight). Pulmonary artery pressure, peak airway pressure, and airway flow were monitored continuously, and lung compliance and PVR were calculated every hour.

### Assessment of endothelial barrier function

After 3 h of EVLP, 6 mg of Evans blue dye (EBD, Millipore Sigma) was administered into the perfusate to assess vascular endothelial permeability^34^. After 30 min of perfusion, the lung vascular bed was rinsed with 20 ml of PBS and pieces of lungs were incubated overnight in formamide (Millipore Sigma) at 60°C. Absorbance of the extractant was measured at 620 nm to quantitate the amount of EBD that had permeabilized into the tissue and was normalized to dry-tissue weight.

### Wet-to-dry (W/D) weight ratio

The weight of the lung tissues was measured immediately after collection (wet weight). The lung tissue was then placed into a 60 °C oven to dry for 72 h. Tissues were weighed to determine the dry weight, and the wet-to-dry ratio was calculated.

### Real-time RT PCR

Lung tissues were collected after EVLP or 2 h after transplantation. Total RNA was extracted from the graft using TRIzol reagent (Life Technologies, Inc. Grand Island, NY) according to the manufacturer’s instructions and purified by ethanol precipitation. RNA content was measured using 260/280 nm UV spectrophotometry. Messenger RNA (mRNA) levels for interleukin (IL)-6, IL-1β, metalloprotease (MMP)-9, and glyceraldehyde-3-phosphate dehydrogenase (GAPDH) were measured with SYBR Green 2-step, real-time reverse transcription-polymerase chain reaction (RT-PCR) as previously described^11^.

### Western blotting

Whole cell lysate was isolated with 2% SDS buffer (2% SDS, 50 mM Tris–HCl pH 6.8, 10% glycerol) supplemented with protease inhibitors (Sigma-Aldrich). Membrane protein fractions were obtained with the Mem-PER Plus Membrane Protein Extraction Kit (Thermo Scientific) following manufacturer’s instructions. Proteins were resolved on 10% SDS-PAGE followed by transfer to 0.2 μm nitrocellulose membranes (Bio-Rad Laboratories, Hercules, CA, USA). Membranes were blocked with 5% non-fat milk at room temperature for 2 h and then incubated with the following primary antibodies at 4 °C overnight: anti-syndecan-1 (1:1000; Santa Cruz Biotechnology, Inc., Dallas, Tx, USA), anti-heparanase-1 (1:1000; Boster Biological, Pleasanton, CA, USA), anti-VE-Cadherin (1:1000; Abcam), anti-HSP90 (1:1000; Abcam) and anti-β-actin (1:50,000; Sigma-Aldrich). Membranes were then incubated with either anti-mouse (1:5000) or anti-rabbit (1:3000) horseradish peroxidase (HRP)-conjugated polyclonal secondary antibody (Abcam, Cambridge, MA, USA), or IRDye 800CW anti-rabbit IgG or 680CW anti-mice secondary antibody (LI-COR Biosciences, Lincoln, NE, USA) at room temperature for 2 h. Proteins were visualized using an enhanced chemiluminescence kit (Abcam), and protein bands were imaged and analyzed using Image Lab software (Version 6.0; Bio-Rad Laboratories, Inc.) or Odyssey 9120 Infrared Imaging System (LI-COR Biosciences).

### Heparanase activity assay

Heparanase activity was measured using a heparanase assay kit (AMS Biotechnology (Europe) Ltd., Abingdon, UK) to quantitate heparan sulfate degradation according to the manufacturer’s instructions. Briefly, isolated lung tissue lysate was applied to biotinylated heparan sulfate-coated wells and incubated with gentle shaking for 1 h at 37°C. After incubation and washing, the remaining heparan sulfate was detected using a biotin-streptavidin-HRP system.

### Determination of glycocalyx components in rat lung grafts

For rat lung tissue, syndecan-1 released into the perfusate during EVLP was measured with a rat-specific ELISA (Immunotag Rat SDC1 (Syndecan-1) ELISA, G-Biosciences, St. Louis, MO). Glycocalyx glycosaminoglycans (GAGs) were quantitated using the 1,9-dimethylmethylene blue (DMMB, Millipore Sigma) method with modifications^35^. A 1 l solution of DMMB (pH ~ 3.0) was prepared by dissolving 16 mg DMMB in distilled H_2_O containing 3.04 g/l glycine, 1.6 g/l NaCl and 95 ml 0.1 M acetic acid. Briefly, 1 ml DMMB solution was added to 20 μl lung tissue lysate then incubated at room temperature for 1 h on a tube rotator. The tubes were then centrifuged at 14,000 rpm for 15 min at 4 °C. Supernatants were carefully decanted, 200 μL sodium lauryl sulfate buffer (2.08 mM) was added to each pellet, and the tubes were vortexed until GAG-DMMB pellets were no longer visible. GAG content in each sample was measured spectrophotometrically at 656 nm.

### Gelatin zymography

Gelatin zymography was performed to measure secreted MMP-2 and MMP-9 activity in EVLP perfusate and to measure endogenous MMP-2 and 9 activity in lung tissue before transplantation. Briefly, lung tissue before transplant was homogenized in cold PBS. Perfusate samples (20 μl) or tissue lysates were mixed with 2× non-reducing SDS sample buffer were loaded onto a 10% polyacrylamide gel containing 0.1% SDS and 0.1% gelatin. After electrophoresis to separate the proteins, the gel was washed in renaturing buffer (Novex Invitrogen) at room temperature, and then incubated in developing buffer (Novex Invitrogen) at 37 °C overnight (~ 16–18 h). The next day, the gel was stained with Coomassie Brilliant Blue R-250 (Bio-Rad) and imaged (ChemiDoc, Bio-Rad). Enzyme activity, visualized as clear bands against the dark blue background, was quantified using Image-J (NIH).

### TEM for glycocalyx imaging

To prepare the tissue for transmission electron microscopy imaging, blood was flushed from rat lungs with PBS via the pulmonary artery after 1 h of warm ischemia, then the lungs were perfused with a solution containing 2% glutaraldehyde, 2% sucrose, 0.1 M sodium cacodylate buffer (pH 7.3, Sigma), and 2% lanthanum nitrate (Sigma). Excised lung tissues were incubated with the same solution for 2 h at 4 °C for fixation. The fixed tissues were immersed in a 2% sucrose, 0.1 M sodium cacodylate buffer (pH 7.3) solution with 2% lanthanum nitrate overnight at 4 °C to stain the glycocalyx, then washed twice with an alkaline solution (0.03 mol/l NaOH) containing 2% sucrose^36^.

Following fixation and glycocalyx staining with lanthanum nitrate, the specimens were rinsed in PBS, post-fixed in 1% osmium tetroxide (Electron Microscopy Sciences, Hatfield, PA), rinsed in PBS a second time, dehydrated through a graded series of ethanol and propylene oxide (Electron Microscopy Sciences), and embedded using a Poly/Bed 812 (Luft formulations) Embedding Kit (Polysciences, Warrington, PA). Sections were cut on a Leica-Reichart Ultracut ultra-microtome (Leica Microsystems, Buffalo Grove, IL). Semi-thin (300 nm) sections were stained with 0.5% toluidine blue in 1% sodium borate (toluidine blue O and sodium borate, Fisher Scientific, Pittsburgh, PA) and examined under the light microscope. Ultrathin sections (65 nm) were stained with uranyl acetate and Reynold’s lead citrate and examined on a JEOL 1400 transmission electron microscope (JEOL Peabody, MA) with a side-mount AMT 2 k digital camera (Advanced Microscopy Techniques, Danvers, MA).

### Tissue staining and histopathological analysis

Formalin-fixed, paraffin-embedded lung tissues collected 2 h after transplantation were sectioned to 4-μm thickness and stained with hematoxylin and eosin. The sections were also stained for immunofluorescent imaging using primary antibodies for CD3 (Invitrogen), CD68 (Invitrogen), or syndecan-1 (Santa Cruz), and Hoechst 33342 dye for nuclear staining. Primary antibody was detected by secondarily Cy5-conjugated goat anti-rabbit IgG (Invitrogen) and Cy3-conjugated goat anti-mouse IgG (Millipore Sigma). Polymorphonuclear neutrophils (PMNs) were stained using a naphthol AS-D chloroacetate esterase staining kit (Millipore Sigma) and identified by nuclear morphology staining in bright red. Stained slides were scanned with a whole-slide image scanner (Axio Scan.Z1; Carl Zeiss) and analyzed with digital image processing software (ZEN lite blue edition; Carl Zeiss). Stained cells were counted by two investigators with the identity of the samples masked.

### Statistical analysis

All data were analyzed using SPSS Version 25 statistical software package (SPSS Inc., Chicago, IL). The data are considered as continuous variables with approximately normal distributions and presented as mean ± standard deviation with individual data plots. The data were analyzed with one-way analysis of variance (ANOVA) followed by post hoc analysis with the Bonferroni correction for multiple comparisons. Data from multiple observations over time were analyzed using 2-way repeated measures ANOVA. A probability level of p < 0.05 was considered statistically significant.

## Supplementary Information

Supplementary Information.

## Data Availability

All data associated with this study are present in the main text or the Supplementary Materials.
