# Supplementary material for: Heparanase inhibition preserves the endothelial glycocalyx in lung grafts and improves lung preservation and transplant outcomes
Source: Sci Rep. 2021 Jun 10;11:12265. doi: 10.1038/s41598-021-91777-0 (PMC8192744; doi:10.1038/s41598-021-91777-0)
Supplement: Supplementary file 1 — Supplementary Information. [file 41598_2021_91777_MOESM1_ESM.pdf]

Supplementary Appendix

Title: Heparanase Inhibition Preserves the Endothelial Glycocalyx in Lung Grafts and Improves Lung Preservation and Transplant Outcomes

Kentaro Noda, Brian J. Philips, Mark E. Snyder, Julie A. Phillippi, Mara Sullivan, Donna B. Stolz, Xi Ren, James D. Luketich, Pablo G. Sanchez.

University of Pittsburgh, Pittsburgh PA

Table of contents

**Contents**	**Page**
Title page for supplemental files	1
**Supplemental figures**	
Figure S1. Representative western blotting for heparanase in lung tissue after ischemic insult	2
Figure S2. Additional transmission electron microscopy images of the glycocalyx.	3
Figure S3. Western blot images to show protein crossover between membrane and cytosol	4
Figure S4. Representative western blotting for syndecan-1.	5
Figure S5. Western blotting for syndecan-1 in membrane and cytosol fractions of the lungs 2 hours after transplantation in multiple samples.	6
Figure S6. Representative images of immunofluorescent staining for syndecan-1 of peripheral airway in lung grafts 2 hours after reperfusion	7
Figure S7. Original whole gel of the gelatin zymography for Figure 5 and replications	8
Figure S8. Gelatin zymography for tissue lysate of pretransplant lungs tissue	9

Supplemental figures

Supplemental figure S1

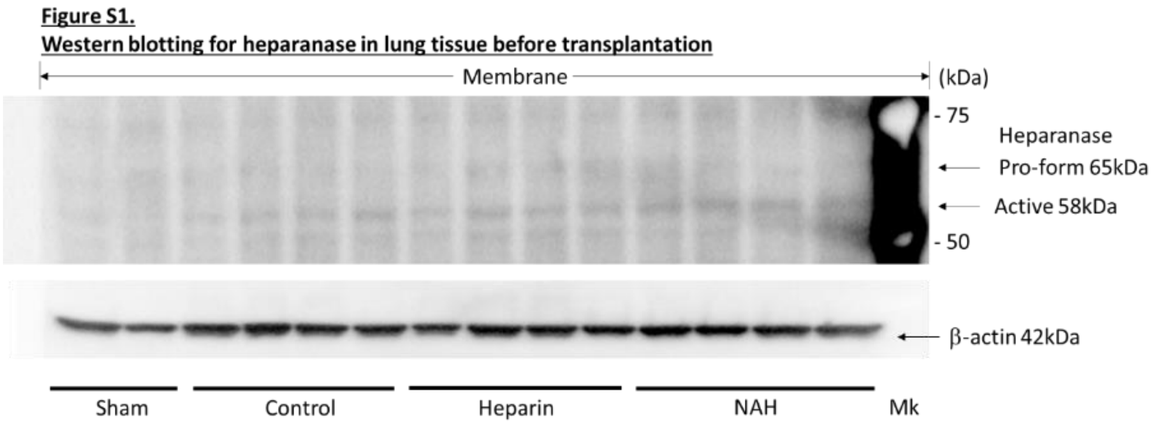

Supplemental figure S1 Full length image of Western blotting for heparanase in the pretransplant lungs tissue with damaged (control) or preserved (heparin and NAH) endothelial glycocalyx. Mk, molecular weight marker.

Supplemental figure S2.

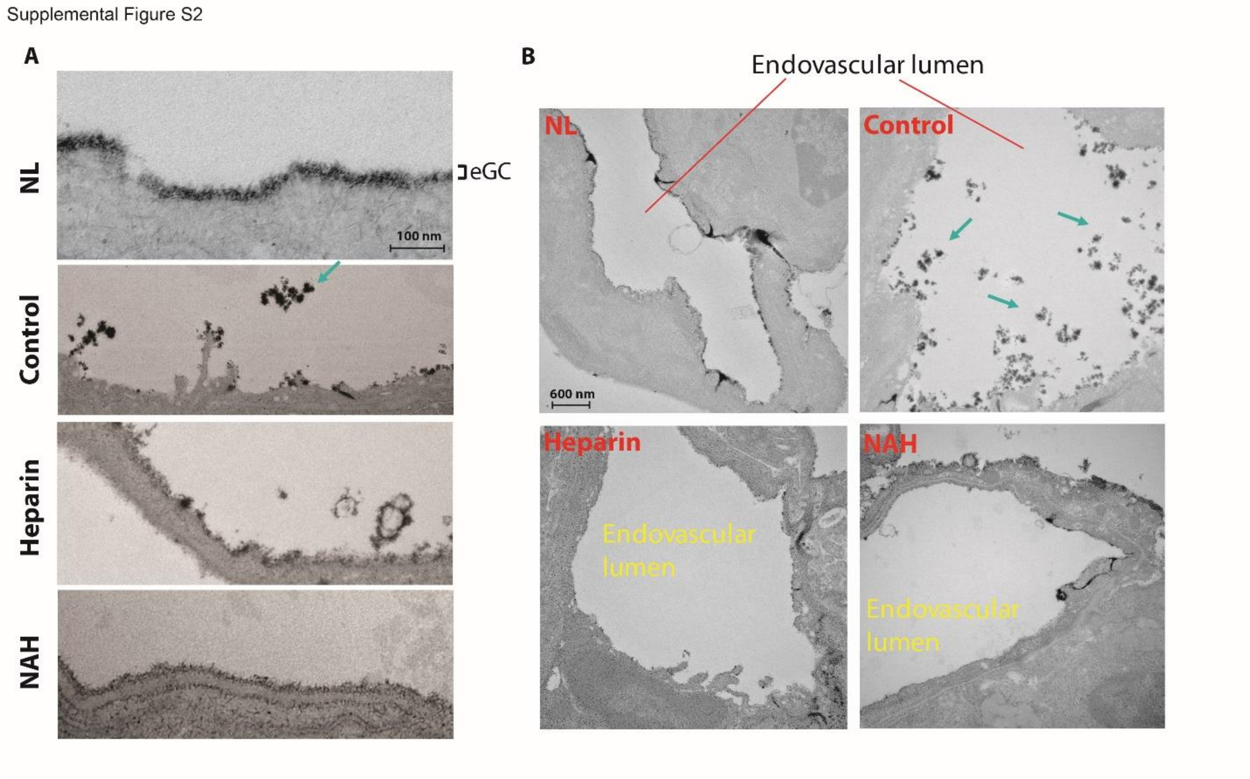

Supplemental figure S2. Supplemental figures for main figure 1C. Additional transmission electron microscopy images of the endothelial glycocalyx (eGC) for each group. (A) Higher magnification of the endothelial cell surface. (B) Endothelial vascular lumen. Arrows indicate glycocalyx shedding. NL, native lungs; Control, control lungs with eGC damage induced by 1 hour of ischemia; Heparin, lungs with eGC preserved by heparin administration prior to 1 hour of ischemia; NAH, lungs with intact eGC preserved by N-acetyl heparin administration prior to 1 hour of ischemia.

Supplemental figure S3.

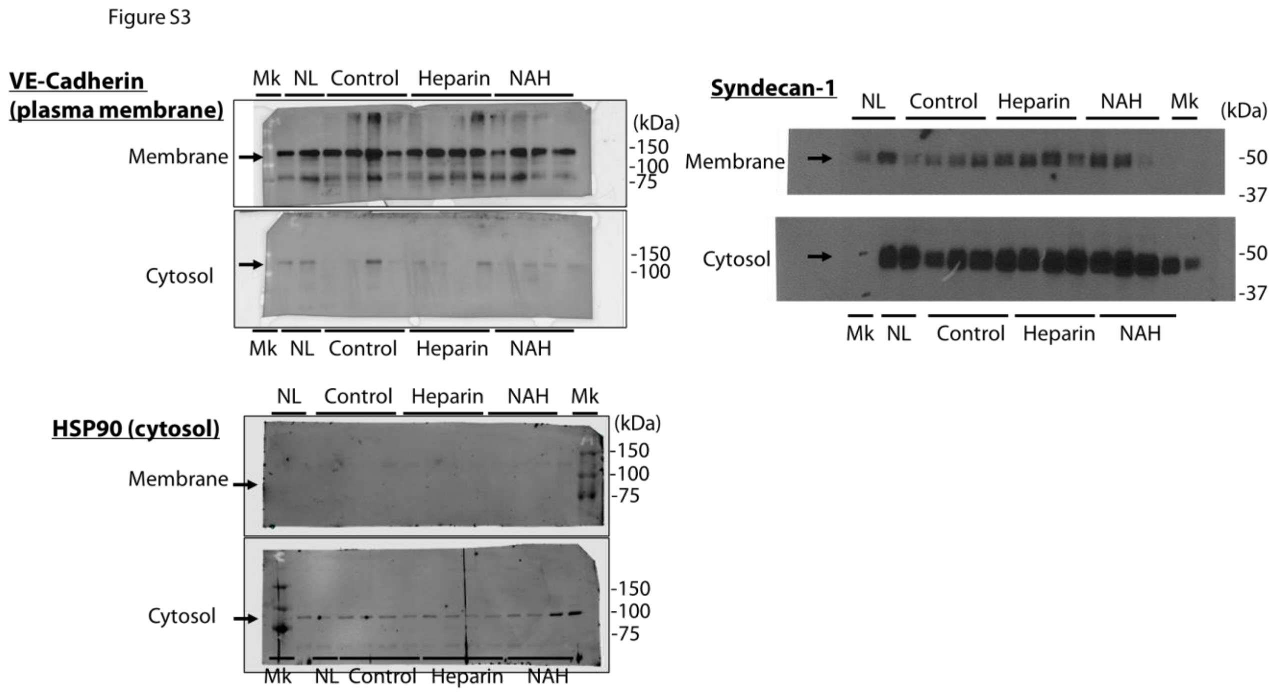

Supplemental figure S3. Supplemental image for figure 4A. Images of full length of western blotting for vascular endothelial (VE)- cadherin, heat shock protein 90 (HSP90), and syndecan-1 to examine membrane and cytosol proteins in all fractionated lung tissue samples collected after transplantation. Membranes were routinely cut between the 75kDa and 50kDa molecular weight markers to assess expression of multiple proteins in the same sample. The images for membrane proteins and cytosol proteins were obtained under the same conditions (e.g. antibody incubation, antibody concentration, blocking time and exposure time) for each blot. Mk, molecular weight marker. NL, native lungs; Control, control lungs with the endothelial glycocalyx (eGC) damage prior to transplant; Heparin, lungs with eGC preserved by heparin prior to transplant; NAH, lungs with eGC preserved by N-acetyl heparin prior to transplant.

Supplemental figure S4.

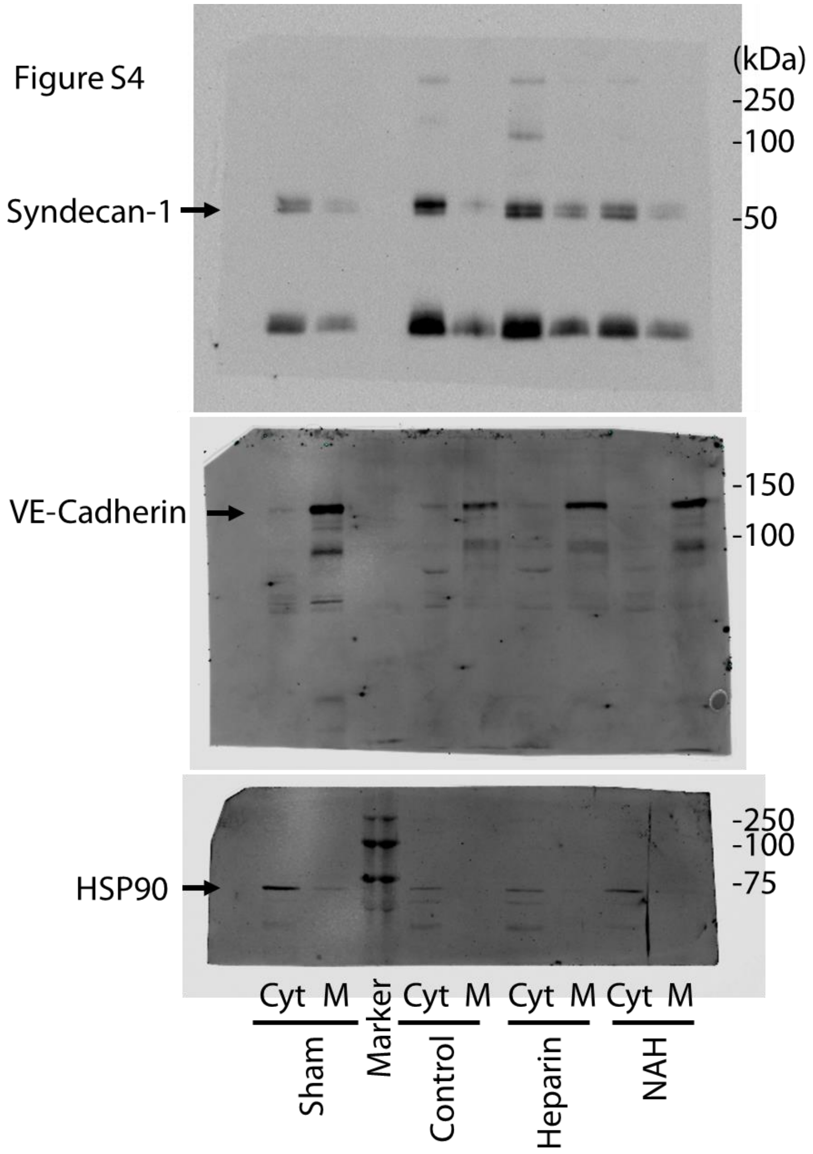

Supplemental figure S4. Supplemental image for figure 4A. Full length images of western blotting for syndecan-1 in membrane and cytosol fractions of single representative sample for each group from the lungs 2 hours after transplantation. The membrane for HSP90 was cut between the 75kDa and 50kDa molecular weight markers to attempt assessment of additional cytoplasmic proteins in the same sample (data not shown). Vascular endothelial (VE)-cadherin and heat shock protein 90 (HSP90) were blotted as loading markers for protein fractions from plasma membrane and cytosol, respectively. Cyt: cytosol protein fraction, M: membrane protein fraction.

Supplemental figure S5.

Figure S5

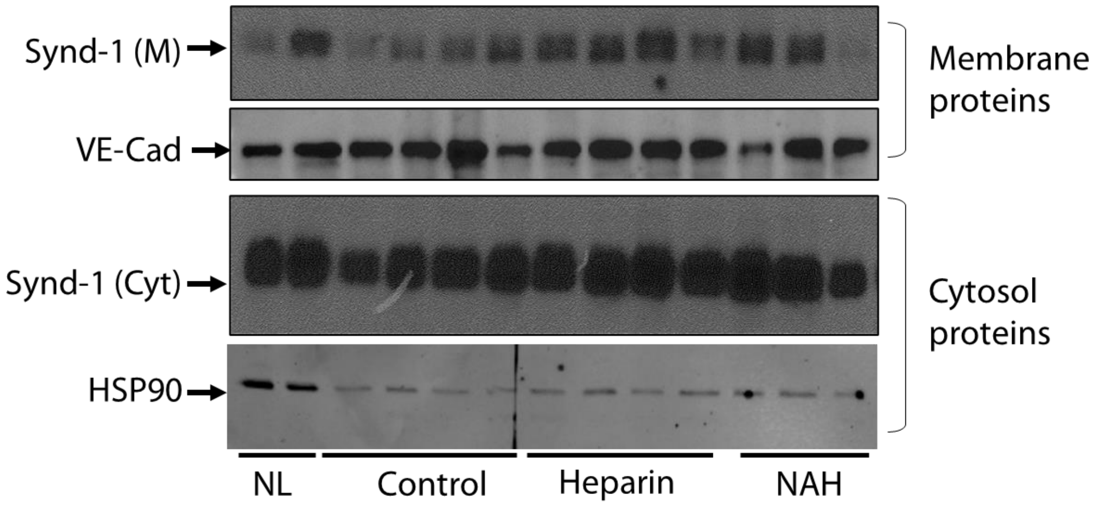

Relative intensity

	NL		Control				Heparin				NAH		
Synd-1/VE-Cad	1.00	1.69	0.72	0.93	0.79	2.74	2.21	1.84	2.48	1.67	5.36	1.77	0.62
Synd-1/HSP90	1.00	1.28	1.22	3.93	4.89	7.43	4.47	4.01	4.39	3.00	3.16	3.28	0.79
Membrane/cytosol Syndecan-1	1.00	1.32	0.59	0.24	0.16	0.37	0.50	0.46	0.57	0.56	1.70	0.54	0.78

Supplemental figure S5. Supplemental image for figure 4A. Quantitation of protein expression in all fractionated lung tissue samples collected 2 hours after transplantation (quantitation of supplemental figure S3). Vascular endothelial cadherin (VE-Cad) and heat shock protein 90 (HSP90) were blotted as loading markers for protein fractions from plasma membrane and cytosol, respectively. Relative intensity of each well was shown below the images. Cyt: cytosol protein fraction, M: membrane protein fraction. NL, native lungs; Control, control lungs with the endothelial glycocalyx (eGC) damage prior to transplant; Heparin, lungs with eGC preserved by heparin prior to transplant; NAH, lungs with eGC preserved by N-acetyl heparin prior to transplant.

Supplemental figure S6.

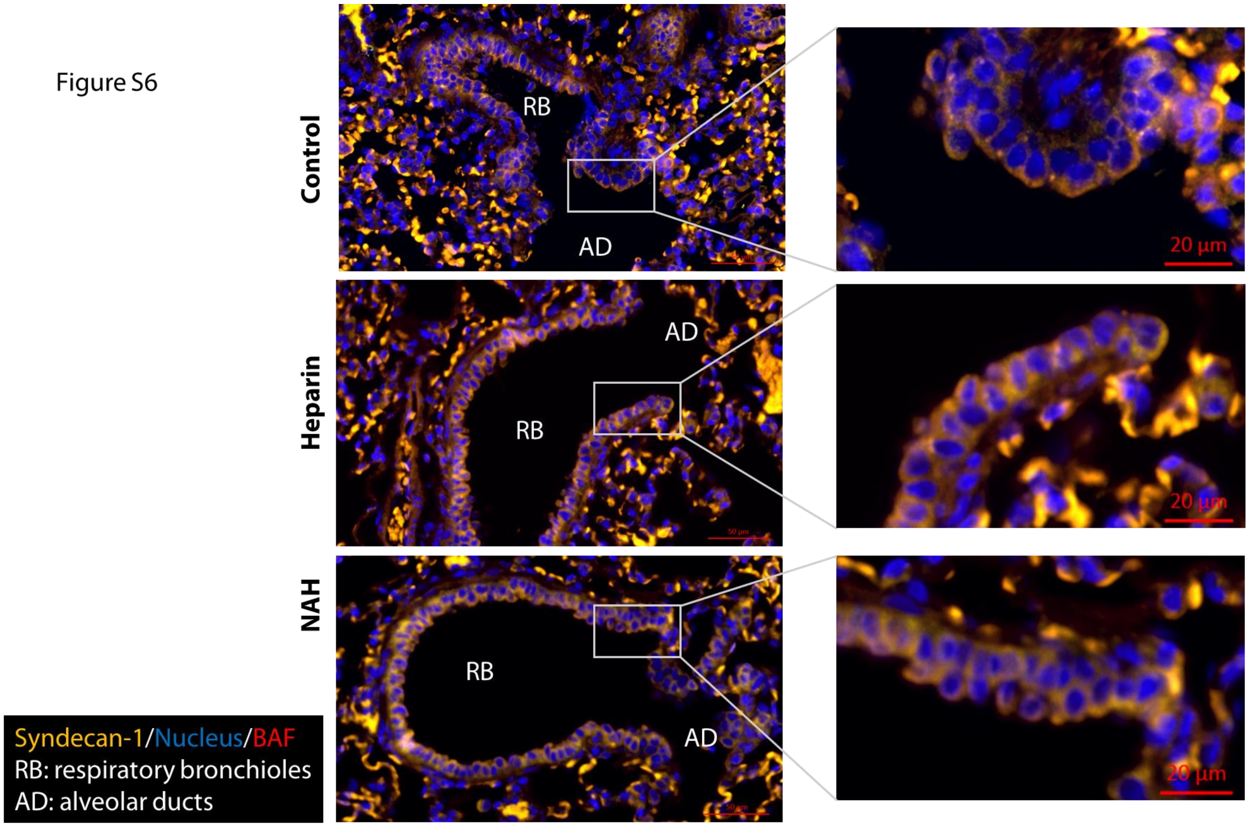

Supplemental figure S6. Supplemental image for figure 4C. Representative images of immunofluorescent staining for syndecan-1 in the peripheral airways in lung grafts 2 hours after transplantation and reperfusion. The region where the respiratory bronchioles (RB) meet the alveolar ducts (AD) was defined as the peripheral airway. Syndecan-1 is visualized in yellow, and nuclei are stained with Hoechst 33342 (shown as blue), and the entire microscopic structure was visualized using background autofluorescence (BAF). Control, experimental model of lung grafts with damaged endothelial glycocalyx (eGC) prior to transplant; Heparin, experimental model of lung grafts with eGC preserved by heparin administration prior to transplant; NAH, experimental model of lungs with eGC preserved by N-acetyl heparin administration prior to transplant.

Supplemental figure S7.

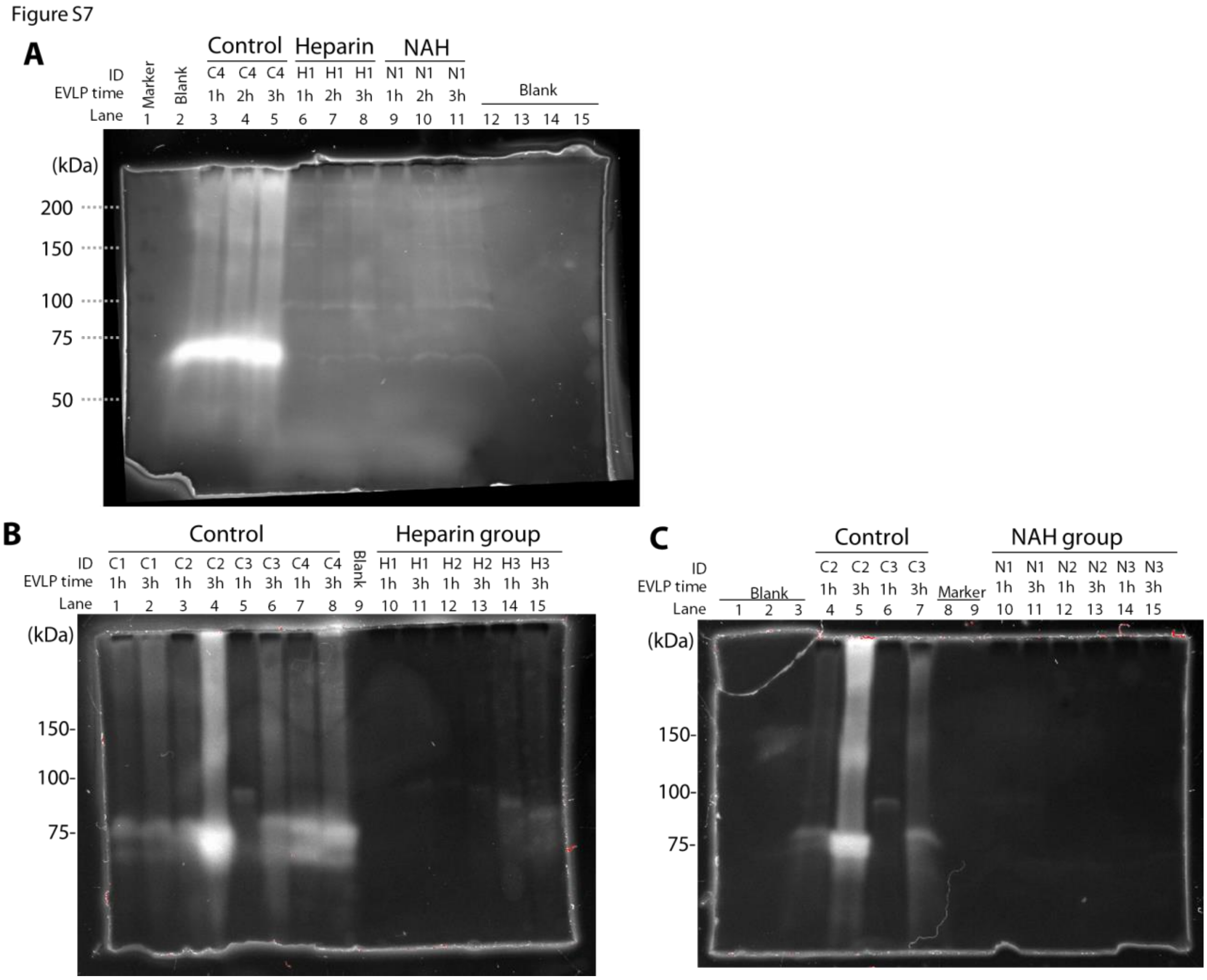

Supplemental figure S7. Supplemental image for figure 5B to show entire gel of the gelatin zymography. (**A**) Original full image of gel for figure 5B. (**B** and **C**) Replication of zymography using EVLP perfusate sampled 1 hour and 3 hours after EVLP initiation. NL, native lungs; Control, control lungs with damage to the endothelial glycocalyx (eGC) prior to transplant; Heparin, lungs with eGC preserved by heparin administration prior to transplant; NAH, lungs with eGC preserved by N-acetyl heparin administration prior to transplant.

Supplemental figure S8.

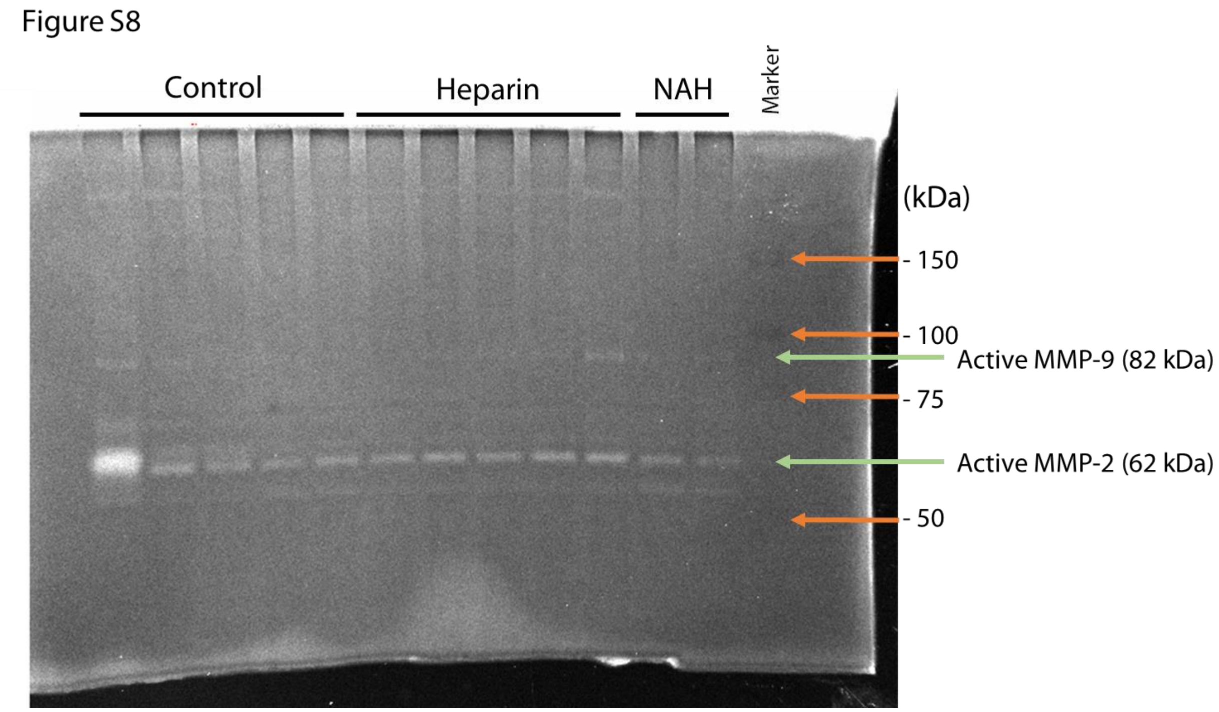

Supplemental figure S8. An image of whole gel of the gelatin zymography for tissue lysate of pretransplant lungs with damaged (control) and preserved (heparin and N-acetyl heparin (NAH)) endothelial glycocalyx to detect endogenic metalloproteinase (MMP)-2 and -9. Endogenous MMP9 expression in lung tissue before transplant/EVLP was clearly lower than MMP2. NL, native lungs; Control, control lungs with the endothelial glycocalyx (eGC) damage prior to transplant; Heparin, lungs with eGC preserved by heparin administration prior to transplant; NAH, lungs with eGC preserved by N-acetyl heparin administration prior to transplant.
